# GWAS and selection signature analyses of whole-genome sequencing data identify genes associated with body size in Duolang sheep

**DOI:** 10.3389/fvets.2026.1869513

**Published:** 2026-07-22

**Authors:** Keyao Wang, Zhigang Niu, Sen Tang, Bing Han, Jie Xu, Yifan Ma, Xiangdong Ding, Hongcai Shi

**Affiliations:** 1 State Key Laboratory of Animal Biotech Breeding, National Engineering Laboratory for Animal Breeding, College of Animal Science and Technology, China Agricultural University, Beijing, China; 2 Key Lab of Reproduction & Breeding Biotechnology of Grass Feeding Livestock of MOA, Xinjiang Academy of Animal Science, Urumqi, China; 3 Henan University of Animal Husbandry and Economy, Zhengzhou, China

**Keywords:** body size traits, Duolang sheep, genome-wide association study, selective sweep analysis, SheepGTEx, whole genome sequencing

## Abstract

**Introduction:**

Duolang sheep is a meat-fat dual-purpose breed originating from Xinjiang, China, and the genetic basis of body size variation in this breed remains unclear.

**Methods:**

Whole-genome resequencing was performed on 224 Duolang sheep with complete records for six body size traits. After variant calling, imputation, and quality control, 19.65 million high-quality SNPs were retained. After linkage disequilibrium pruning, 2.1 million relatively independent SNPs were used for a mixed linear model GWAS in GEMMA. Tajima’s D and integrated haplotype score analyses were performed in a kinship-filtered subset of 60 animals, and SheepGTEx data were used to characterize tissue expression patterns.

**Results:**

The GWAS identified 24 significant SNPs for height, body length, chest girth, shoulder width, and hip width. Integration of GWAS intervals with selection-signal regions prioritized 31 candidate genes, including *CA10*, *WDR45B*, *FADS2*, *LGALS12*, and *CTNNA2*. Multi-tissue expression profiles provided functional context for the prioritized genes.

**Discussion:**

These findings provide insight into the genetic architecture of body size traits in Duolang sheep and identify candidate loci for future validation and genetic improvement studies.

## 1 Introduction

Sheep are vital livestock species with a widespread distribution ranging from the Tibetan Plateau to eastern China. Based on geographic distribution and genetic relationships, Chinese sheep populations are generally classified into three primary lineages: Tibetan, Mongolian, and Kazakh (55). Among these, Duolang sheep, a distinct indigenous meat breed in northern Xinjiang, is renowned for its large body size, rapid growth rate, and superior meat quality, characterized by tenderness, evenly distributed fat, and a distinctive flavor with reduced gaminess (1). These attributes help meet the local demand for mutton. However, industrial expansion and shifting dietary preferences have created a supply–demand gap in Xinjiang’s mutton market. Clarifying the genetic basis of body size traits in Duolang sheep is therefore important for understanding breed-specific conformation and for developing candidate markers that support genetic improvement.

With the rapid advancement of sequencing technologies, genome-wide association studies (GWAS) based on whole-genome sequencing have become important for investigating the genetic basis of complex traits. Compared with SNP array data, whole-genome sequencing provides a denser marker resource and can capture variants across the genome more comprehensively. However, sequence-based GWAS also introduces challenges related to data quality, marker redundancy, multiple testing, and computational burden. Mixed linear models, such as GEMMA (2), are widely used to account for population structure and relatedness. More recently, efficient algorithms such as BOLT-LMM (3), REGENIE (4), and SAIGE (5) have been developed for large-scale association studies. Functional annotation scores, such as CADD (6), can help prioritize variants predicted to have potential functional effects. Multiple-testing correction methods, including FDR (7) and Bonferroni correction (8), are also commonly used to control false-positive associations.

In recent years, the sharp drop in the cost of obtaining dense genomic markers has made GWAS a mainstream approach for pinpointing genetic variants underlying economically important traits (9). In sheep, body size is a crucial indicator of growth, health, and environmental adaptability (10, 11). Extensive GWAS have been deployed to unravel the genetic architecture of these traits. For instance, numerous significant loci and candidate genes (e.g., *RAB6B* and *GIGYF2*) have been mapped for early growth traits, such as birth and weaning weights, across various local breeds (12–14). Furthermore, genomic profiling has successfully identified specific genes driving broader mature body size (e.g., *GRID1*, *STX8*, and *SMARCA5*) and related metabolic traits (15, 16).

Despite the undeniable power of GWAS in dissecting the genetic architecture of complex traits, it primarily captures variants that are still segregating within a population. However, livestock breeds have historically experienced intense artificial and natural selection for economically important traits, such as body size and environmental adaptability. Long-term selection leaves distinct genomic footprints, including reduced diversity and extended linkage disequilibrium. To detect these signatures, statistics like Tajima’s D (17) and the integrated haplotype score (iHS) (18) serve as complementary approaches. Integrating GWAS with selection signature analysis can therefore provide overlapping evidence for prioritizing candidate loci involved in phenotypic variation and breed formation. For instance, this integrated framework has been successfully applied to decipher the polygenic architecture of economically important traits in various livestock species, such as carcass traits in beef cattle (19) and body weights in chickens (20).

In this study, whole-genome resequencing data were used to characterize genomic variation in Duolang sheep. An LD-pruned tag-SNP-based GWAS was integrated with genome-wide selection signature analyses to identify candidate loci for body size traits. Multi-tissue transcriptomic data from SheepGTEx were further used to examine the tissue expression patterns of prioritized candidate genes and provide tissue-level functional context (21).

## 2 Materials and methods

### 2.1 Animals and phenotypic data

A total of 224 healthy adult Duolang sheep with complete phenotypic records were included from the National Duolang Sheep Conservation Farm of Wuzheng Company (Makit County, Kashgar Prefecture, Xinjiang, China). The 224 animals consisted of 134 males and 90 females. All individuals were born around February 2022. On March 17, 2023, six body size traits were measured, including height at withers, body length, chest girth, shoulder width, hip width, and chest depth. In accordance with animal ethics and welfare guidelines, blood samples were collected from the ear vein of each individual and used for whole-genome resequencing on the Illumina NovaSeq6000 PE150 platform.

### 2.2 Variant calling and filtering

Raw reads were trimmed using Trimmomatic v0.39 (22) and aligned to the *Ovis aries* reference genome (oviAri4) using BWA-MEM v0.7.17 (23). BAM files were sorted and indexed with SAMtools v1.10 (24), and PCR duplicates were marked using Sambamba v0.9.5 (25). The mean genome-wide sequencing depth across the 224 sequenced individuals was 1.12×, ranging from 1.02 × to 1.17×. Variant discovery and joint genotyping were performed for all individuals using GATK HaplotypeCaller v4.1.9 in GVCF mode (26). The resulting variants were filtered using GATK VariantFiltration and BCFtools v1.10.2 (24) to retain only biallelic SNPs meeting the following criteria: QD ≥ 2.0, MQ ≥ 40.0, FS ≤ 60.0, SOR ≤ 3.0, MQRankSum ≥ − 12.5, and ReadPosRankSum ≥ − 8.0. Genotype imputation was performed using Beagle v5.2 (27) with an internal reference panel of 40 Duolang sheep sequenced at approximately 10x depth. Imputation accuracy was evaluated using masking validation, and the overall accuracy reached 91.73%. The imputed variants were further filtered using BCFtools v1.10.2 to retain biallelic SNPs with a minor allele frequency (MAF) ≥ 0.01 and per-site missingness ≤ 10%. After imputation and quality control, 19.65 million high-quality SNPs were retained for downstream population structure and selection signature analyses.

### 2.3 Population genetic structure analysis

Population structure was assessed using principal component analysis (PCA) and linkage disequilibrium (LD) decay. For PCA, SNPs were pruned for linkage disequilibrium in PLINK v1.90 (28) using the command --indep-pairwise 50 10 0.7. PCA was used to characterize the genetic structure among the sequenced individuals and to define population clusters for subsequent kinship-based sampling in the selection signature analysis. Pairwise LD decay was calculated using PopLDdecay v3.41 (29) with parameters -MaxDist 300-MAF 0.005-Het 0.5. All figures were generated using R v4.3.5.

### 2.4 Genome-wide association study

A tag-SNP-based GWAS for the six body size traits was performed in the 224 sequenced Duolang sheep with complete phenotypic records using a mixed linear model implemented in GEMMA v0.98 (2). The model was specified as follows:


y=Xα+Zβ+Wμ+e

Where y represents the vector of phenotypic values; α denotes the vector of fixed effects, including the top 3 principal components (PCs) to account for population structure; β indicates the effect size of the SNP being tested; μ signifies the vector of random polygenic effects, following a multivariate normal distribution $\mu \sim N(0,GV_{g})$ , where G is the genomic relationship (kinship) matrix and $V_{g}$ is the additive genetic variance; e is the vector of random residuals, following a distribution $e\sim N(0,IV_{e})$ , where I is the identity matrix and $V_{e}$ is the residual variance; and W , X , and Z are the corresponding incidence matrices for α , β , and μ , respectively.

After LD pruning in PLINK v1.90 (28), 2,103,847 relatively independent tag SNPs were retained for association testing. This tag-SNP set was used to reduce marker redundancy, lower the multiple-testing burden, and improve computational tractability in a relatively small WGS-based population. Therefore, the association analysis should be interpreted as a tag-SNP-based GWAS rather than a full association scan of all 19.65 million sequence-derived variants. To control for population stratification and cryptic relatedness, the first three PCs were included as fixed covariates, and a centered genomic relatedness matrix (GRM) was incorporated as a random effect. Genome-wide significance was determined using the Benjamini-Hochberg false discovery rate (FDR) method, with FDR correction performed separately for each trait and a significance threshold of *q* < 0.05.

### 2.5 Selective sweep analysis

Based on the population structure revealed by PCA, the Duolang sheep were divided into three PCA-defined clusters. To maintain balanced representation across clusters and reduce genetic redundancy, a core subset of 60 sequenced individuals was constructed by selecting 20 individuals from each cluster based on kinship estimates. Within each cluster, individuals with relatively low pairwise relatedness were preferentially retained for selective sweep analysis. The 60 selected individuals were then analyzed as a single core subset for within-population selection-signal analysis. Tajima’s D was calculated using VCFtools v0.1.16, and iHS was computed using selscan v1.3.0 (30) to detect genomic regions under selection. Genotype phasing for iHS was performed using Beagle v5.2 (27) with default phasing settings before running selscan. The selection-signal results were integrated with GWAS loci to prioritize candidate regions for body size traits.

Tajima’s D values were calculated in non-overlapping 50-kb windows using the 19.65 million high-quality SNPs. Windows corresponding to the top and bottom 1% of Tajima’s D values were considered candidate regions under selection. The iHS test was applied to the same high-quality SNP set to identify haplotype-based selection signals after genotype phasing. Genotype phasing was performed using Beagle v5.2 with default parameters. Selscan v1.3.0 was run in iHS mode using phased VCF files with a --max-gap setting of 200,000 bp. Raw iHS scores were standardized using the norm utility distributed with selscan. After normalization, loci with |iHS| > 3 were considered extreme iHS signals.

### 2.6 Candidate gene prioritization and enrichment analysis

Candidate genomic regions were defined by integrating GWAS loci with selection-signal regions. For GWAS, significant SNPs were extended ± 150 kb upstream and downstream to form candidate intervals. For selection signatures, regions identified by Tajima’s D were extended ± 150 kb, whereas |iHS| intervals were extended ± 25 kb. All protein-coding genes overlapping these expanded regions were extracted using the BioMart tool from Ensembl (*Ovis aries* release 106).^1^

To investigate the functional relevance of candidate genes, Gene Ontology (GO) enrichment and Kyoto Encyclopedia of Genes and Genomes (KEGG) pathway analyses were performed using the Database for Annotation, Visualization and Integrated Discovery (DAVID) v6.8 (31). Candidate gene sets were obtained from the overlap between GWAS signals and selection scan results (e.g., Tajima’s D and iHS). GO terms and KEGG pathways with *p* < 0.05 were considered statistically significant.

### 2.7 Tissue expression profiling of candidate genes using SheepGTEx

To characterize the tissue expression patterns of candidate genes, expression data for seven genes (*CA10*, *ADRB2*, *WDR45B*, *FADS1*, *FADS2*, *LGALS12*, and *CTNNA2*) were retrieved from the Sheep Gene Tissue Expression Atlas (SheepGTEx) (21), which provides transcriptomic profiles across 60 tissues in sheep. Expression levels were quantified as transcripts per million (TPM), and median TPM values were calculated for each gene across all tissues. To normalize the skewed distribution of expression values, a log2(TPM + 1) transformation was applied prior to downstream analyses.

Expression profiles were visualized using heatmaps generated across all available tissues and a subset of body size-related tissues (including musculoskeletal, adipose, endocrine, and nervous system tissues). Hierarchical clustering was performed using Ward’s minimum variance method with Euclidean distance to identify co-expression patterns among genes and tissue groupings. Tissue-specific expression distributions were further examined using violin plots combined with box plots for key tissues relevant to growth and body composition.

Tissue specificity of each candidate gene was quantified using the TAU index (32). The TAU index ranges from 0 (ubiquitous expression) to 1 (highly tissue-specific expression), with a threshold of 0.8 used to classify genes as tissue-specific. To assess co-expression relationships among the seven candidate genes, pairwise Spearman rank correlation coefficients were computed based on their median expression values across all 60 tissues.

To provide a systems-level overview of candidate gene expression, the 60 tissues were grouped into 10 functional categories: musculoskeletal, adipose, digestive, cardiovascular, nervous, endocrine, reproductive, integumentary, immune, and urinary. Mean expression levels within each category were visualized using a dot plot. In addition, a tripartite network diagram was constructed to summarize the relationships among body size traits with GWAS signals, their associated candidate genes, and the five tissues showing the highest expression levels for each gene, providing tissue-level context for candidate gene prioritization.

## 3 Results

### 3.1 Descriptive statistics of body size traits

The phenotypic statistics for body size traits of Duolang sheep are presented in Table 1. The mean values for height, body length, chest girth, shoulder width, hip width, and chest depth were 75.92, 72.08, 89.45, 19.22, 22.05, and 29.89 cm, respectively. The coefficients of variation ranged from 6.14 to 10.61%, with height showing the lowest variation and shoulder width showing the highest variation. The frequency distributions of the six body size traits are shown in Supplementary Figure 1.

**Table 1:** Descriptive statistics of body size traits in Duolang sheep.

Item	Height	Body length	Chest girth	Shoulder width	Hip width	Chest depth
Mean	75.92	72.08	89.45	19.22	22.05	29.89
Maximum	88	94	109	24	29	36
Minimum	60	53	71	13	14	20
SD	4.66	4.96	6.82	2.04	2.2	2.46
Coefficient of variation, %	6.14	6.88	7.62	10.61	9.98	8.23

### 3.2 Population structure analysis

**Figure 1:**
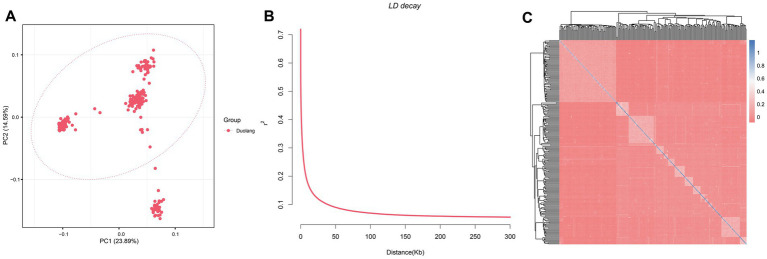
Population structure and relatedness of Duolang sheep. **(A)** PCA distribution of the sequenced Duolang sheep. The ellipse indicates the 95% confidence ellipse. **(B)** Linkage disequilibrium decay pattern. **(C)** Heatmap of the kinship matrix for Duolang sheep.

The PCA based on the 224 sequenced Duolang sheep revealed moderate population stratification and relatedness (Figure 1A), with the first two PCs collectively explaining 38.48% of the total genetic variance. All 224 sequenced individuals had complete phenotypic records and were included in the GWAS. To minimize false-positive associations, the top three PCs were included as fixed-effect covariates, and a centered kinship matrix was fitted as a random effect. As shown in Figure 1C, the average kinship coefficient was −0.0045, with localized blocks indicating cryptic family structures. This approach was used to reduce confounding due to population structure and family relatedness.

Linkage disequilibrium decay patterns further characterized the population’s evolutionary history, with pairwise r^2^ values declining to background levels (r^2^ < 0.1) within 40 kb physical distances (Figure 1B). This rapid LD decay suggests a relatively high recombination rate and weak long-range linkage, consistent with historically large effective population size and limited recent artificial selection pressure.

### 3.3 Genome-wide association study

**Figure 2:**
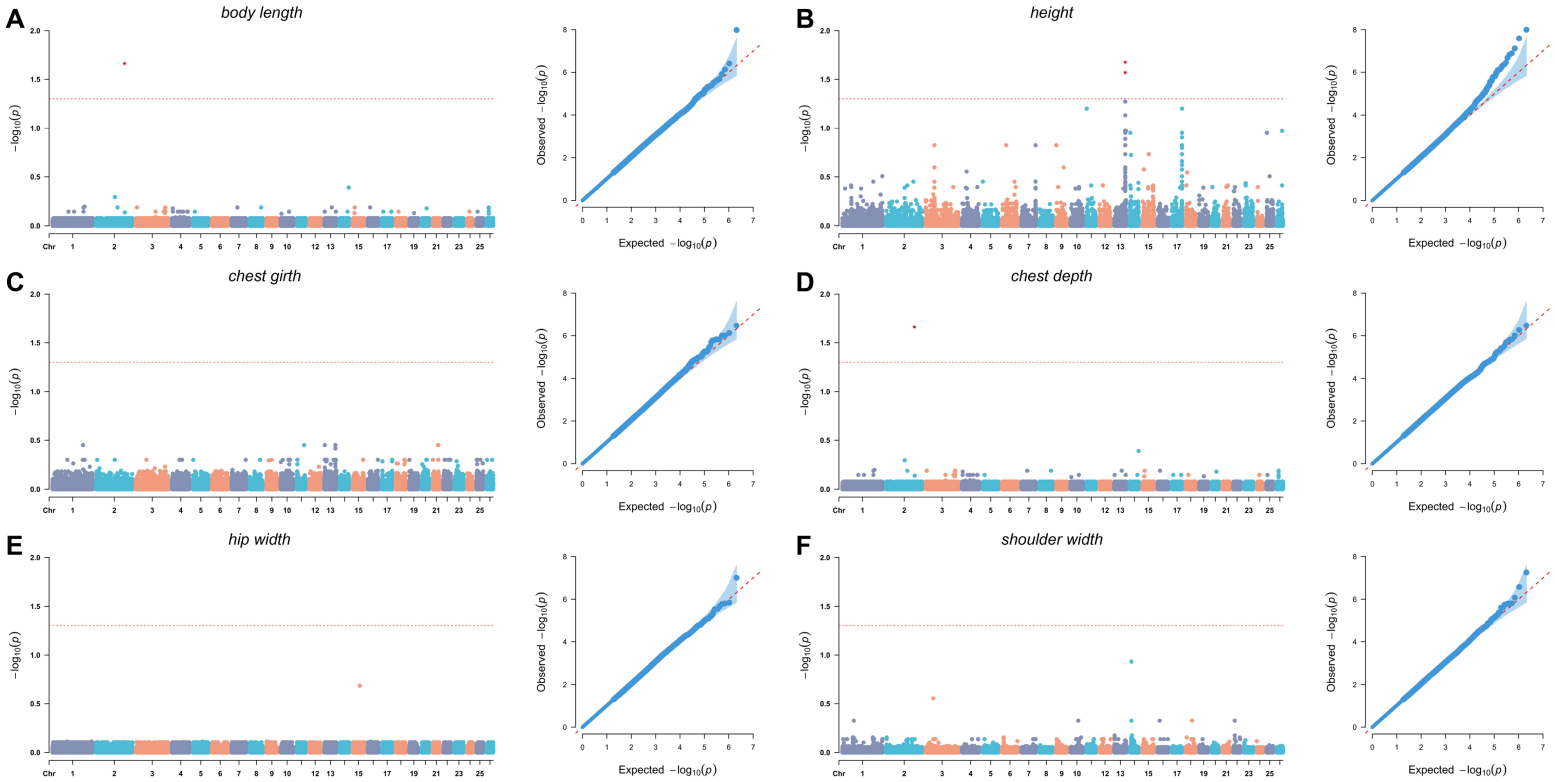
Manhattan plots and Q-Q plots for GWAS of body conformation traits. **(A)** Body length. **(B)** Height. **(C)** Chest girth. **(D)** Chest depth. **(E)** Hip width. **(F)** Shoulder width.

A tag-SNP-based GWAS was conducted using a mixed linear model in GEMMA software to analyze six body size traits (height, body length, chest girth, shoulder width, hip width, and chest depth) in the 224 sequenced Duolang sheep. Significant SNPs were detected for height, body length, chest girth, shoulder width, and hip width, whereas no significant associations were detected for chest depth (Figure 2).

Specifically, a total of 24 significant SNPs were identified across the analyzed traits (Supplementary Table 1). For body height, 10 associated SNPs were mapped to Chr 11, 13, and 17. Similarly, 10 SNPs associated with chest girth were located on Chr 1, 11, 13, and 21. For the remaining traits, fewer signals were detected: two SNPs for shoulder width (Chr 3 and 14), one for body length (Chr 2), and one for hip width (Chr 15).

### 3.4 Selective sweep analysis

**Figure 3:**
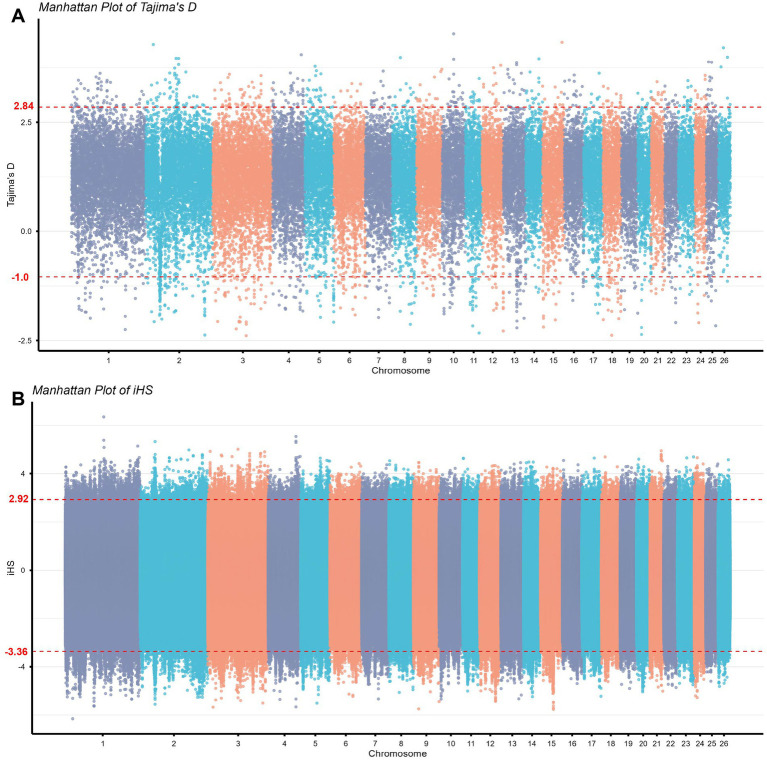
Selective sweep analysis of 60 Duolang sheep. **(A)** Manhattan plot of Tajima’s D values. **(B)** Manhattan plot of iHS values.

The distributions of Tajima’s D windows and iHS loci across the genome are shown in Figure 3. Tajima’s D and iHS were both calculated using the 19.65 million high-quality SNPs retained after imputation and quality control. Based on the top and bottom 1% of Tajima’s D scores, 968 candidate windows were identified, including 484 windows with high Tajima’s D values and 484 windows with low Tajima’s D values. Genes located within the extended Tajima’s D candidate intervals were collected for overlap analysis with GWAS loci, resulting in 2,036 genes. Extreme iHS signals were defined using normalized iHS scores with |iHS| > 3, and genes located within the extended iHS candidate intervals were collected, resulting in 2,951 genes. A total of 530 genes were shared between the Tajima’s D and iHS candidate gene sets. These selection-signal results were integrated with GWAS candidate intervals to identify overlapping genes for downstream prioritization.

### 3.5 Identification of candidate genes

We examined the overlap between genes located in GWAS candidate intervals and genomic regions showing selection signatures to prioritize candidate genes for body size traits in Duolang sheep. A total of 56 gene records were annotated within the GWAS candidate intervals. Among these, 31 genes overlapped with regions showing Tajima’s D or iHS selection signals and were considered candidate genes jointly supported by GWAS and selection signature analyses (Table 2; Supplementary Table 2). Based on literature evidence, seven of these genes (*CA10*, *ADRB2*, *WDR45B*, *FADS1*, *FADS2*, *LGALS12*, and *CTNNA2*) were further highlighted because they have been reported to participate in growth, skeletal development, adipose deposition, metabolism, or other biological processes potentially related to body size traits.

**Table 2:** Selected significant GWAS SNPs and representative nearby candidate genes for body size traits.

Traits	Chr	Position, bp	*p* value	Candidate gene
Height	13	75,234,631	1.01E-08	*SLC35C2*
Height	11	111,103	1.29E-07	*CA10*
Height	13	75,225,895	3.56E-07	*OCSTAMP*
Height	17	70,290,615	1.29E-07	*SFI1*
Body length	2	194,783,980	1.04E-08	*ENSOARG00020065679*
Chest girth	13	75,255,859	3.36E-07	*OCSTAMP*
Chest girth	1	208,981,198	1.57E-06	*ADRB2*
Chest girth	11	49,559,585	9.91E-07	*WDR45B*
Chest girth	21	36,788,255	1.47E-06	*FADS1*
Shoulder width	14	10,211,673	1.69E-06	*WFDC1*
Shoulder width	3	51,520,020	2.65E-07	*CTNNA2*
Hip width	15	48,782,627	9.83E-08	*OR52E4*

### 3.6 Functional enrichment analysis

**Figure 4:**
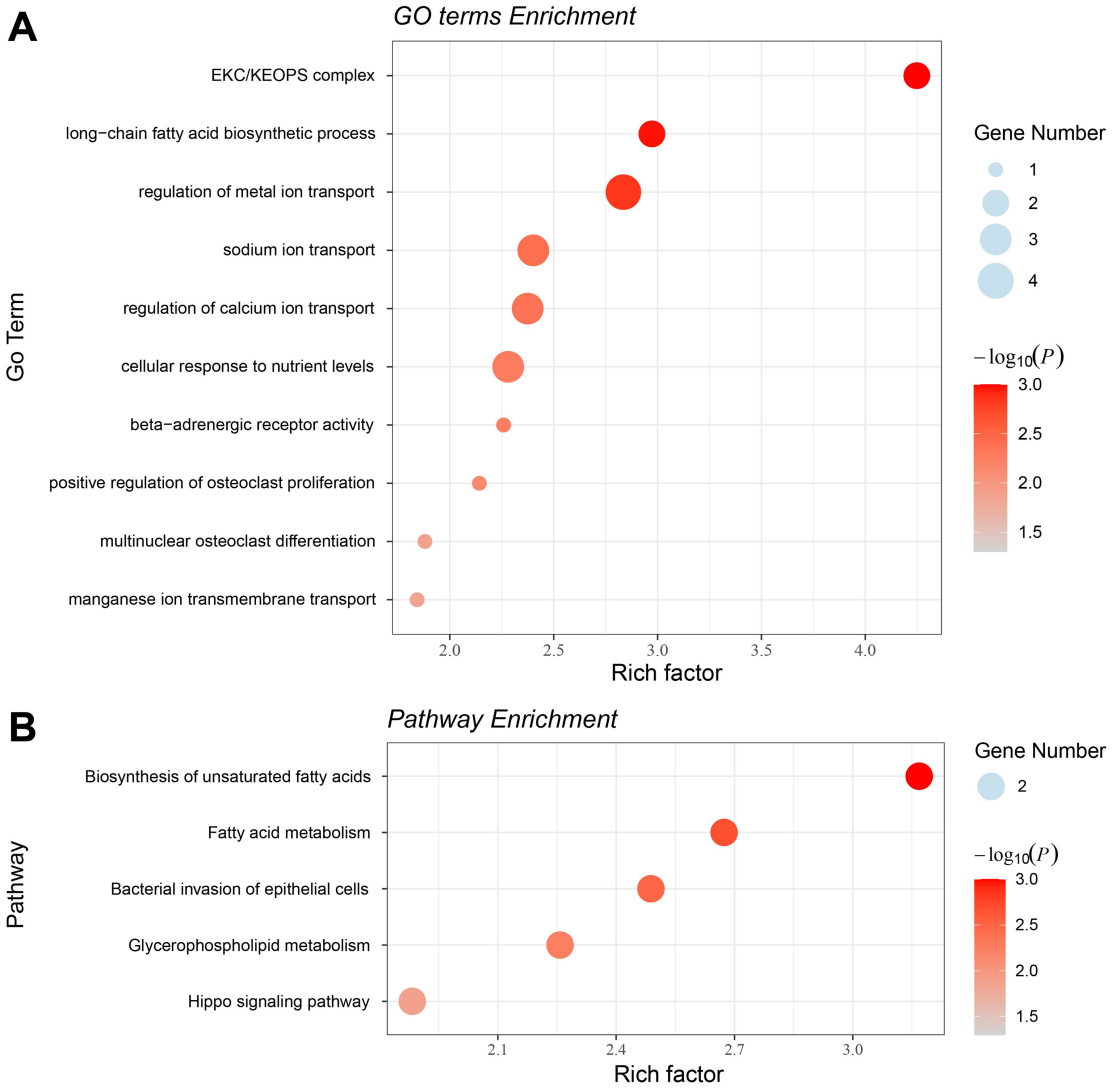
GO and KEGG enrichment analysis. **(A)** GO enrichment terms for the overlap genes. **(B)** KEGG pathways reported for functional interpretation.

To investigate the biological functions of the candidate genes, functional enrichment analysis was performed (Figure 4). In the Biological Process (BP) category, these genes were mainly enriched in terms related to growth and development, such as regulation of calcium ion transport, positive regulation of osteoclast proliferation (33), and long-chain fatty acid biosynthetic process. Additionally, they were functionally associated with beta-adrenergic receptor activity (34) and the EKC/KEOPS complex (35) in the molecular function and cellular component categories, respectively. KEGG pathway analysis highlighted the potential involvement of these genes in the Hippo signaling pathway (36), which regulates organ size, as well as lipid-related pathways including biosynthesis of unsaturated fatty acids and fatty acid metabolism (37)(Table 3).

**Table 3:** Functional enrichment analysis of candidate genes.

Categories	Term	*p* value
KEGG_PATHWAY	biosynthesis of unsaturated fatty acids	0.02
KEGG_PATHWAY	Hippo signaling pathway	0.07
GOTERM_BP_DIRECT	regulation of calcium ion transport	0.01
GOTERM_BP_DIRECT	positive regulation of osteoclast proliferation	0.01
GOTERM_BP_DIRECT	long-chain fatty acid biosynthetic process	0.01
GOTERM_MF_DIRECT	beta-adrenergic receptor activity	0.01
GOTERM_BP_DIRECT	EKC/KEOPS complex	0.01

### 3.7 Multi-tissue transcriptomic expression annotation of candidate genes

**Figure 5:**
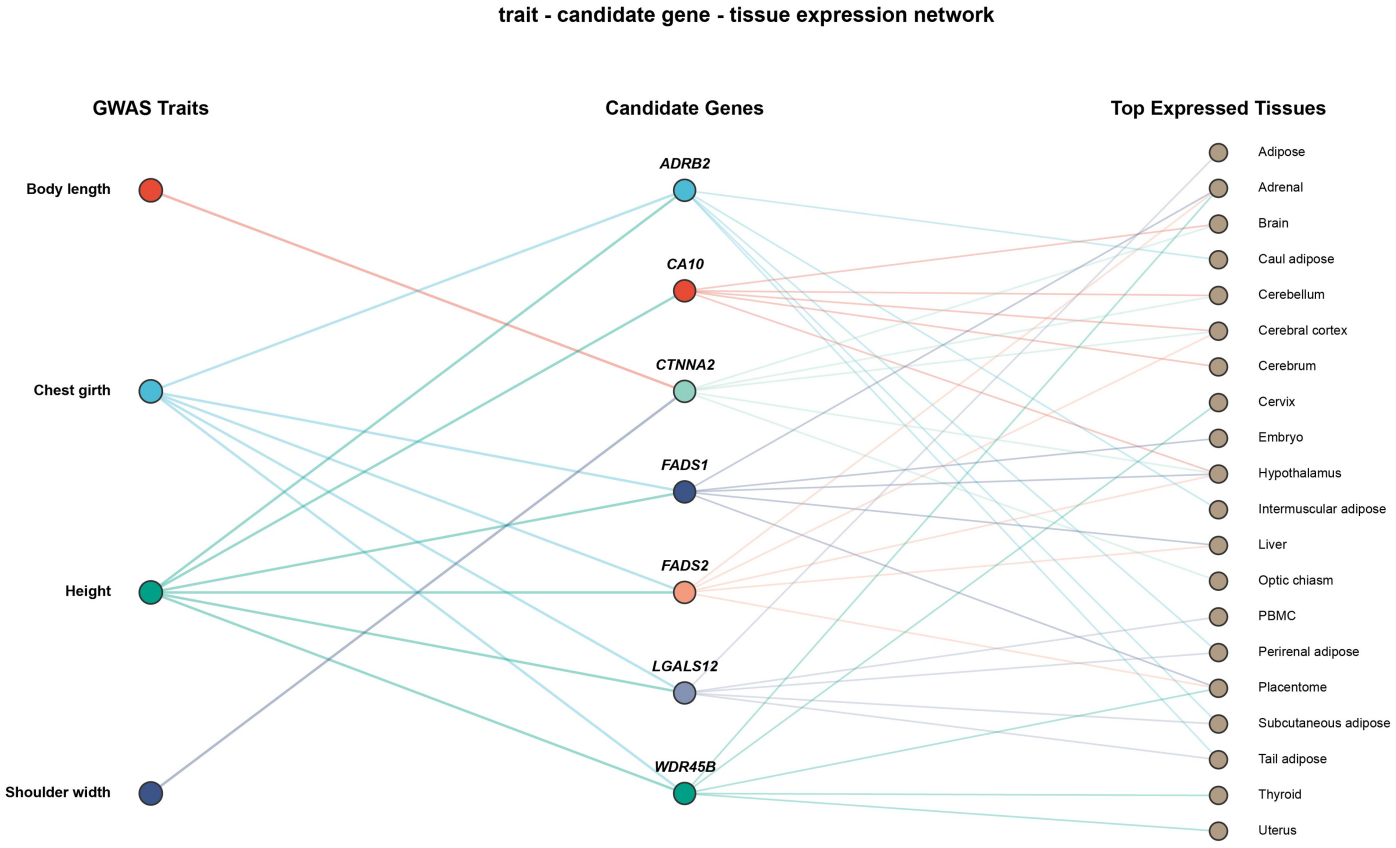
Trait-candidate gene-tissue expression annotation network in Duolang sheep.

To summarize the relationships among body size traits, candidate genes, and tissues, a trait-gene-tissue expression annotation network was constructed (Figure 5). This network connected GWAS-supported traits with prioritized genes and the tissues in which these genes showed relatively high expression. Height was linked to candidate genes including *CA10*, *ADRB2*, *WDR45B*, *FADS1*, *FADS2*, and *LGALS12*, with expression profiles spanning nervous, adipose, and metabolic tissues. Chest girth was linked to *ADRB2*, *WDR45B*, *FADS1*, *FADS2*, and *LGALS12*, whereas body length and shoulder width were connected to *CTNNA2*. These patterns provide functional context for candidate prioritization and highlight tissues relevant to growth and body conformation.

**Figure 6:**
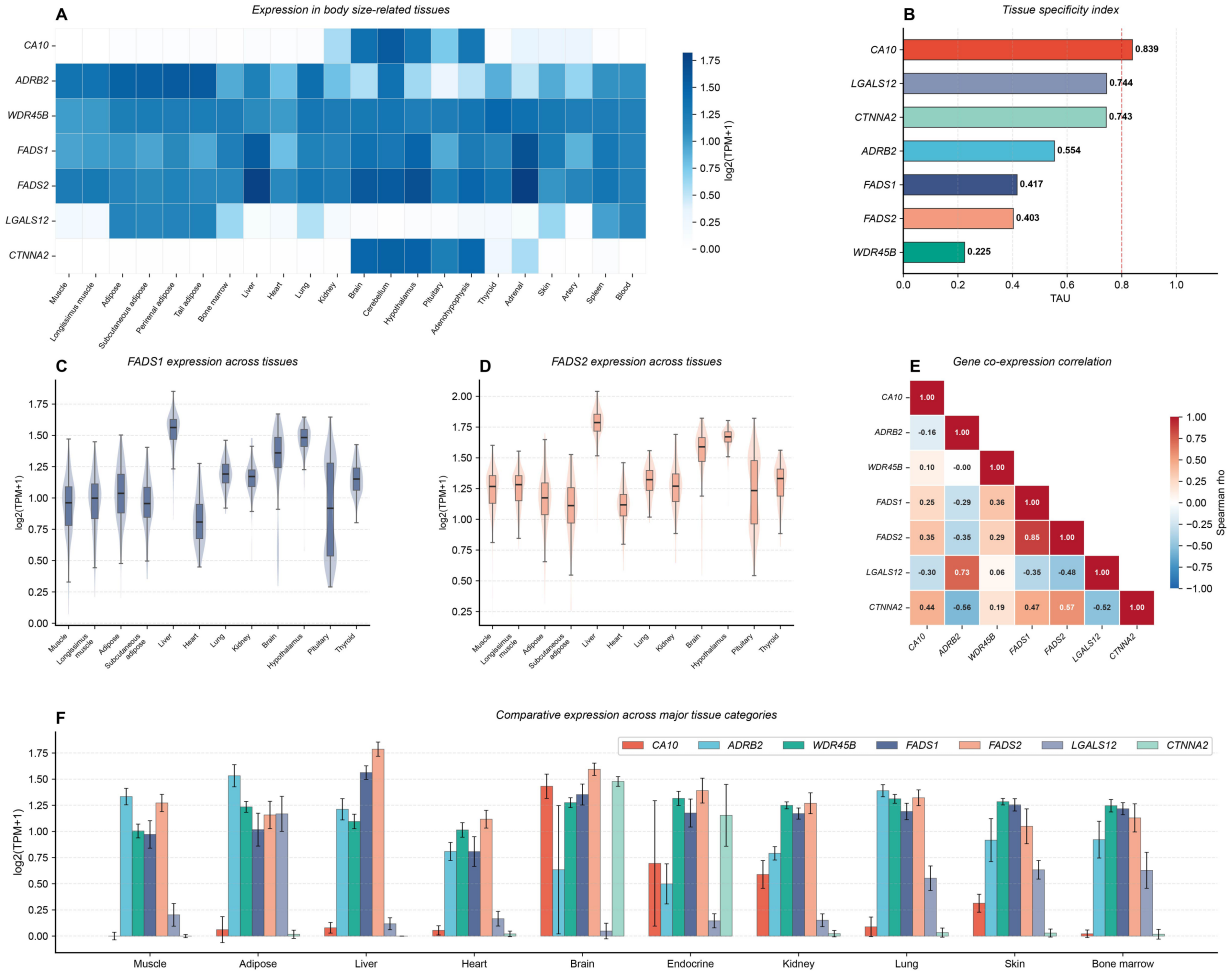
Multi-tissue transcriptomic profiling of the candidate genes. **(A)** Heatmap illustrating expression patterns of candidate genes across 60 SheepGTEx tissues. **(B)** Tissue specificity index (TAU) of the candidate genes. **(C,D)** Violin plots showing expression distributions of FADS1 **(C)** and FADS2 **(D)** across selected body size- and metabolism-related tissues. **(E)** Gene expression correlation matrix based on multi-tissue expression profiles. **(F)** Comparative expression profiles of the candidate genes grouped into 10 functional tissue categories.

Based on transcriptomic data from the SheepGTEx database, the expression patterns of the seven candidate genes were examined. The global expression heatmap (Figure 6A) showed three broad expression patterns: a nervous system-enriched pattern (*CA10*, *CTNNA2*), an adipose tissue-enriched pattern (*ADRB2*, *LGALS12*), and a broader metabolic/endocrine pattern (*WDR45B*, *FADS1*, *FADS2*). The tissue specificity index (TAU) quantification (Figure 6B) showed that *CA10* had high tissue specificity (TAU = 0.839), followed by *LGALS12* (TAU = 0.744) and *CTNNA2* (TAU = 0.743). *ADRB2*, *FADS1*, and *FADS2* showed moderate tissue biases (TAU = 0.554, 0.417, and 0.403, respectively), while *WDR45B* displayed lower specificity (TAU = 0.225). Comparative analysis across 10 major anatomical categories (Figure 6F) showed that *ADRB2* had relatively high expression in the adipose category, whereas *FADS2* showed broad expression across multiple anatomical categories.

Violin plots were used to describe the sample-level expression distributions of metabolism-related genes within tissues. *FADS1* and *FADS2* showed relatively high expression distributions in the liver, hypothalamus, and several endocrine tissues (Figures 6C,D). Multi-tissue Spearman correlation analysis (Figure 6E) further revealed expression similarities among candidate genes across tissues. The strongest positive correlation was observed between *FADS1* and *FADS2* (rho = 0.85), followed by *ADRB2* and *LGALS12* (rho = 0.73), consistent with their similar tissue expression patterns. In contrast, negative correlations, such as those between *ADRB2* and *CTNNA2* (rho = −0.56) and between *LGALS12* and *CTNNA2* (rho = −0.52), indicated distinct tissue expression profiles among genes potentially involved in different biological processes.

## 4 Discussion

Population stratification and cryptic relatedness can lead to inflated test statistics and spurious associations in GWAS (38). PCA is commonly used to capture major axes of genetic variation (39), while mixed linear models account for relatedness through a kinship or genomic relationship matrix (40, 41). Because population structure analysis revealed genetic differentiation within the Duolang sheep population, we used an MLM framework that included the first three PCs as fixed covariates and a centered genomic relatedness matrix as a random effect. This approach helped reduce confounding from both population structure and relatedness.

It should be noted that the association analysis in this study was based on an LD-pruned tag-SNP set rather than on all sequence-derived variants. Although 19.65 million high-quality SNPs were retained after imputation and quality control, 2,103,847 relatively independent tag SNPs were used for GWAS after LD pruning. Tag-SNP strategies can reduce marker redundancy, decrease the multiple-testing burden, and improve computational feasibility, but they may also reduce mapping resolution when causal or rare variants are not well tagged by the retained markers (42, 43). Therefore, the significant SNPs identified in this study should be interpreted primarily as tag-SNP association signals that help define candidate genomic intervals rather than as exhaustive causal variants from a full-marker WGS association scan.

In addition, the current GWAS model did not include several non-genetic covariates, such as sex, exact age, birth type, body weight at measurement, or measurement batch. These factors are commonly considered in livestock association studies because they may influence growth and body size traits (44, 45). In the present population, all animals were born within a similar period, raised under the same conservation-farm management conditions, and measured on the same date, which likely reduced variation caused by age, environment, and measurement batch. However, both males and females were included, with 134 males and 90 females. Therefore, although population structure and relatedness were controlled using the first three PCs and a centered genomic relatedness matrix, the absence of sex and other detailed covariates should be considered when interpreting the GWAS results. Future association analyses with larger sample sizes, full-marker testing, and more complete covariate records will help refine these loci and improve the robustness of the detected signals.

### 4.1 Candidate genes related to skeletal development and neuroendocrine regulation

Longitudinal growth and structural traits (e.g., height and shoulder width) are fundamentally driven by bone metabolism and central developmental signaling. *OCSTAMP* (Osteoclast Stimulatory Transmembrane Protein) plays a pivotal role in this process by regulating the fusion and differentiation of osteoclasts, which is essential for bone remodeling and longitudinal growth (46). Its genetic influence on skeletal dimensions is evolutionarily conserved; meta-analyses in humans have identified *OCSTAMP* variants as key determinants of adult height and skeletal frame size (47), and similar associations with hip height have been validated in cattle (48).

Concurrently, *CA10* (Carbonic Anhydrase X) was considered a candidate gene potentially related to body size. Although it encodes a catalytically inactive protein, nucleotide variations in *CA10* have been associated with femoral bone mineral density, suggesting a role in bone development and size regulation (49). Furthermore, *CA10* functions as a conserved neurexin ligand in the central nervous system, implicating it in developmental signaling networks that could influence somatic growth through neuroendocrine pathways (50). Additionally, *CTNNA2*, identified here for shoulder traits, belongs to the *CTNNA* gene family known for regulating somatic growth (50). As structural proteins are crucial for cellular integrity and tissue architecture (51), *CTNNA2* may contribute to shoulder development in Duolang sheep by regulating cytoskeletal dynamics and cell–cell adhesion during musculoskeletal morphogenesis.

### 4.2 Candidate genes related to metabolism

Rapid physical expansion requires substantial metabolic and biochemical support. *FADS1* (fatty acid desaturase 1) was identified as a candidate gene potentially related to lipid metabolism. It encodes the delta-5 desaturase, a rate-limiting enzyme required for the synthesis of long-chain polyunsaturated fatty acids (LC-PUFAs) and their downstream signaling lipids. In mammals, desaturase activity is tightly linked to membrane lipid homeostasis and metabolic regulation, providing an essential biochemical basis for rapid somatic growth. In sheep, nutritional and management regimens have been shown to alter *FADS1* expression together with changes in tissue fatty acid profiles (37). Alongside lipid remodeling, fundamental cellular homeostasis is equally crucial for sustaining rapid tissue expansion. In our study, *WDR45B* (WD repeat domain 45B) was also highlighted among metabolism-related candidates. As a ubiquitously expressed gene, *WDR45B* is fundamentally involved in autophagy and intracellular trafficking—processes vital for maintaining cellular integrity and metabolic turnover during periods of high physiological demand (52). The identification of both *FADS1* and *WDR45B* suggests that lipid metabolism and cellular homeostasis may contribute to body conformation in Duolang sheep.

### 4.3 Candidate genes related to adipose deposition

The distinct physical conformation of Duolang sheep is reflected in local fat deposition and muscle development, a process that may involve *LGALS12* (Galectin 12) and *ADRB2* (adrenoceptor beta 2). Identified as a candidate gene for chest girth, *LGALS12* is predominantly expressed in adipose tissue and has been implicated in adipocyte differentiation and lipolysis (53). In sheep, genomic scans for selection signatures have reported *LGALS12* within regions associated with growth rate and carcass traits (54). *ADRB2* was also identified as a candidate for chest girth. Because *ADRB2* is involved in adrenergic signaling related to lipid mobilization and muscle physiology, it provides a plausible biological link to chest conformation.

Together, the GWAS, selection-signal overlap, and tissue expression profiles provide complementary evidence for prioritizing candidate genes related to body size traits in Duolang sheep. This integrated framework helps connect SNP-level signals with biological pathways involved in skeletal development, lipid metabolism, adipose deposition, and tissue growth.

Together, the tag-SNP-based GWAS, selection-signal overlap, and tissue expression profiles provided complementary evidence for prioritizing candidate genes related to body size traits in Duolang sheep.

## 5 Conclusion

By integrating whole-genome sequencing data from Duolang sheep with an LD-pruned tag-SNP-based GWAS and selection signature analyses, this study identified several candidate genes for body size traits, including *CA10*, *CTNNA2*, *OCSTAMP*, *LGALS12*, *FADS1*, and *FADS2*. Expression profiles from SheepGTEx provided additional functional context, suggesting that these candidates may be related to skeletal morphogenesis, neural signaling, lipid metabolism, and adipose deposition. These results enrich the genomic resources available for Duolang sheep and provide candidate markers for future genetic improvement studies after further validation.

## Data Availability

The data presented in the study are deposited in the NCBI Sequence Read Archive (SRA) under BioProject accession number PRJNA1475921.

## Footnotes

1
http://asia.ensembl.org/biomart

## Supplementary material

Supplementary Table 1 Significant SNPs identified in the genome-wide association studies for body size traits.

Supplementary Table 2 Gene sets used for the integration of GWAS and selection-signal analyses, including selection-signal genes, GWAS candidate genes, and overlapping candidate genes.

Supplementary Figure 1 Frequency distributions of six body size traits in the 224 sequenced Duolang sheep used for GWAS: height, body length, chest girth, shoulder width, hip width, and chest depth.
