# Supplementary material for: GWAS and selection signature analyses of whole-genome sequencing data identify genes associated with body size in Duolang sheep
Source: Front Vet Sci. 2026 Jul 22;13:1869513. doi: 10.3389/fvets.2026.1869513 (PMC13437492; doi:10.3389/fvets.2026.1869513)
Supplement: Supplementary file 1 [file Supplementary_file_1.docx]

**Supplementary Figure 1.** Frequency distributions of body size traits in Duolang sheep.

The histograms show the phenotypic distributions of height at withers, body length, chest girth, shoulder width, hip width, and chest depth in 224 Duolang sheep.
